# Supplementary material for: Transcriptional blood signatures for active and amphotericin B treated visceral leishmaniasis in India
Source: PLoS Negl Trop Dis. 2019 Aug 16;13(8):e0007673. doi: 10.1371/journal.pntd.0007673 (PMC6713396; doi:10.1371/journal.pntd.0007673)
Supplement: S1 Table — (PDF) [file pntd.0007673.s003.pdf]

S1 Table. Demographic and clinical details of subjects used in the study.

	Experiment 1			Experiment 2		
	Active VL Multidose Amphotericin B	Treated Cases Multidose Amphotericin B	Healthy Controls*	Active VL Single Dose Ambisome	Treated VL Single Dose Ambisome	Healthy Controls*
N (post-QC)	10	10	16	11	11	25
Age (years)	31.0±12.6	31.0±12.6	40.9±17.8	21.6±9.4	30.7±7.7	24.8±17.6
Sex % (M:F)	78:22	78:22	44:56	68:32	65:35	36:64
Weight (Kg)	43.6±7.6	44.3±7.86	51.8±9.8	40.4±9.7	41.5±9.7	40.7±13.3
Platelets (x10^3 U/L)	145±106	235±43	173±46	138±52	275±140	189±82
WBC (cells/ul)	3347±2922	6238±2655	10488±1561	3827±1072	9209±2805	10988±2651
Haemoglobin (g/dl)	9.4±1.7	9.6±1.1	11.8±2.5	8.2±1.7	9.8 ±1.2	12.6±1.5
Lymphocyte (% of WBC)	479±17	40±9	31±6	52±10	35±10	34±9
Eosinophils (% of WBC)	4.8±1.6	3.6±1.1	5.8±0.8	1.4±0.7	1.5±0.7	5.4±0.7
Neutrophils (% of WBC)	45±19	55±10	61±6	43±10	59±9	58±9
Monocyte (% of WBC)	1.2±0.4	1.6±0.8	2.1±0.8	4.1±0.8	4.2±0.4	1.9±0.7
Creatinine (mg/dL)	0.8±0.1	1.2±0.5	ND	0.7±0.2	0.6±0.1	ND

*Healthy controls comprise: 2 antibody positive (both with DAT titer >1:25,600), 8 quantiferon positive (all with DAT titer <1:800), and 6 negative controls (all with DAT titer <1:800) in experiment 1; and 6 antibody positive (all with DAT titer >1:25,600) , 9 quantiferon positive (all with DAT titer <1:800), and 10 negative controls (all with DAT titer <1:800) in experiment 2. Abbreviations: WBC = White Blood Cells. †Mean value ± SD of aggregated data are shown, N/D= not done.
