# Supplementary material for: Transcriptional blood signatures for active and amphotericin B treated visceral leishmaniasis in India
Source: PLoS Negl Trop Dis. 2019 Aug 16;13(8):e0007673. doi: 10.1371/journal.pntd.0007673 (PMC6713396; doi:10.1371/journal.pntd.0007673)
Supplement: S2 Table — Based on expression values for all genes comparing uninfected endemic healthy controls (EHC) with asymptomatic individuals who were positive in antigen-specific assays for high DAT titre antibody levels (high DAT+) or interferon-γ levels in the modified quantiferon test (IFN+). Provides a summary of the number of gene sets that were enriched in each phenotype compared to the Blood Transcription Module (BTM) gene list for antibody responses to vaccines [2] or the GSEA-MSigDB C2 [1] (C2CP) gene list. Results are shown for FDR cut-offs of 0.25 and 0.05. No gene lists were concordant across two experiments for the comparison of EHC with IFN+ asymptomatics. The comparison of DAT+ asymptomatics with either EHC or IFN+ asymptomatics could only be determined in experiment 2 (DAT+ for experiment 1 was N = 2 which could not be analysed in GSEA). (PDF) [file pntd.0007673.s004.pdf]

S2 Table. Results of rank-based nonparametric Gene Set Enrichment Analysis (GSEA [1]) based on expression values for all genes comparing uninfected endemic healthy controls (EHC) with asymptomatic individuals who were positive in antigen-specific assays for high DAT titre antibody levels (high DAT^+) or interferon-γ levels in the modified quantiferon test (IFN^+). Provides a summary of the number of gene sets that were enriched in each phenotype compared to the Blood Transcription Module (BTM) gene list for antibody responses to vaccines [2] or the GSEA-MSigDB C2 [1] (C2CP) gene list. Results are shown for FDR cut-offs of 0.25 and 0.05. No gene lists were concordant across two experiments for the comparison of EHC with IFN^+ asymptomatics. The comparison of DAT^+ asymptomatics with either EHC or IFN^+ asymptomatics could only be determined in experiment 2 (DAT^+ for experiment 1 was N=2 which could not be analysed in GSEA).

		BTM		BTM		C2P2		C2P2	
		FDR<0.25		FDR<0.05		FDR<0.25		FDR<0.05	
Phenotype 1 (P1)	Phenotype 2 (P2)	Enriched P1	Enriched P2	Enriched P1	Enriched P2	Enriched P1	Enriched P2	Enriched P1	Enriched P2
Experiment 1									
IFN^+	EHC	0	0	0	0	0	0	0	0
Experiment 2									
IFN^+	EHC	4	13	0	0	80	0	0	0
DAT^+	EHC	18	22	1	3	0	3	0	0
DAT^+	IFN^+	0	0	0	0	0	4	0	0

References

1. Subramanian A, Tamayo P, Mootha VK, Mukherjee S, Ebert BL, Gillette MA, et al. Gene set enrichment analysis: a knowledge-based approach for interpreting genome-wide expression profiles. Proc Natl Acad Sci U S A. 2005;102(43):15545-50. doi: 10.1073/pnas.0506580102. PubMed PMID: 16199517; PubMed Central PMCID: PMCPMC1239896.
2. Li S, Rouphael N, Duraisingham S, Romero-Steiner S, Presnell S, Davis C, et al. Molecular signatures of antibody responses derived from a systems biology study of five human vaccines. Nat Immunol. 2014;15(2):195-204. Epub 2013/12/18. doi: 10.1038/ni.2789. PubMed PMID: 24336226; PubMed Central PMCID: PMCPMC3946932.
