# Supplementary material for: Transcriptional blood signatures for active and amphotericin B treated visceral leishmaniasis in India
Source: PLoS Negl Trop Dis. 2019 Aug 16;13(8):e0007673. doi: 10.1371/journal.pntd.0007673 (PMC6713396; doi:10.1371/journal.pntd.0007673)
Supplement: S3 Table — Provides details of the BTM gene lists that were significant at FDR<0.05 for the comparisons of DAT+ with EHC in experiment 2. NES = normalized enrichment score which adjusts for gene set size. Negative NES values indicate that the first listed phenotype in column 1 is negatively correlated with the gene set. As indicated by results presented in the main text, none of the genes that contribute to these gene lists, whether contributing to BTM or C2P2 genes lists at FDR<0.25 or FDR<0.05, were significant at B-H adjusted p-values <0.05 in the Limma analysis. They are therefore unlikely to be of value as biomarkers for the DAT+ or IFN+ phenotypes without further detailed longitudinal studies. (PDF) [file pntd.0007673.s005.pdf]

S3 Table. Results of rank-based nonparametric Gene Set Enrichment Analysis (GSEA) as for S2 Table. Provides details of the BTM gene lists that were significant at FDR<0.05 for the comparisons of DAT^{+} with EHC in experiment 2. NES = normalized enrichment score which adjusts for gene set size. Negative NES values indicate that the first listed phenotype in column 1 is negatively correlated with the gene set. As indicated by results presented in the main text, none of the genes that contribute to these gene lists, whether contributing to BTM or C2P2 genes lists at FDR<0.25 or FDR<0.05, were significant at B-H adjusted p-values <0.05 in the *Limma* analysis. They are therefore unlikely to be of value as biomarkers for the DAT^{+} or IFN^{+} phenotypes without further detailed longitudinal studies.

Phenotype comparison	Name of Gene List	Size of gene lists	NES	Nominal p-value	FDR q-value	Leading edge genes contributing to the enriched gene list
DAT^{+} *vs* EHC	T cell activation and signalling (M5.1)	23	1.84	0.004	0.01	GIMAP6, CD3E, TRA@, IL12RB1, CCR7, LCK, TRAT1, CD3D, ZAP70, ITK, CD3G, IL2RG, CD2, TRAF3IP3, PRKACB, HLA-DRB1
EHC *vs* DAT+	Enriched in monocytes (II) (M11.0)	176	-1.73	0.010	0.037	CPPED1, CSF3R, FAM198B, SIRPA, CEBPD, TYMP, NRGN, ITGAX, LMO2, BTK, PRKCD, SYK, CTSH, RNF130, CHST15, PADI2, FCGR2A, LILRB3, SCPEP1, MTMR11, CD14, TLR8, SERPINA1, FPR1, SLC31A2, NCF1C, S100A9, LILRA2, RBM47, AQP9, PYGL, LST1, CSF1R, IFI30, FPR2, VNN2, CLEC4A, KIAA0513, ZNF467, IGSF6, MNDA, TIMP2, SECTM1, MS4A7, SLC15A3, TYROBP, CST3, CYBB, MEGF9, PRAM1, PILRA, RXRA, S100A8, SLC7A7, SEPX1, CPVL, IL13RA1, LY86, RASSF4, CLEC4E, PTAFR, MPEG1, FCGR1A, TBXAS1, RTN1, LILRA5, EPB41L3, FCGRT, TGFBI, PLBD1, FES, IRAK3, CYFIP1, TNS3, FBP1, P2RY13, SULF2, PLAUR, MAFB, CRISPLD2, GRN, CD302, F13A1, HK3, SORT1, CD36, HPSE, PLXNB2, TREM1, CD33, ADAP2, CSTA, GAS2L1, IMPA2, S100A12, NCF1, RAB32, NOD2, CDA, ALDH2, HNMT, VNN3, KIAA1598, CD86, CD93, ASGR1, PLXDC2, NLRP3, CEBPA, ANPEP, BST1, KCTD12, NLRP12, CLEC7A, KYNU, CSF2RA, ASGR2, TMEM176A, SIGLEC9, RIN2, MS4A6A, ALDH1A1, MS4A4A, LRP1, TLR5, CYP1B1, TLR2, RBP7, SIGLEC7, CD163, CACNA2D3, MGST1, LGALS2, KCNE3, CCR1, STAB1, FXYD6, SIRPB1, PLA2G7
	Myeloid cell enriched receptors and transporters *M4.3)	29	-1.70	0.002	0.043	CHST15, MTMR11, CD14, TLR8, LRRC25, CLEC4A, IGSF6, SECTM1, SLC15A3, AMICA1, SLC7A7, SLC24A4, MPEG1, GPBAR1, SULF2, CD86, ASGR1, RNASE6, KCTD12, MS4A14, SLC8A1, TLR5, SIGLEC7, CCR1, MARCO
	Enriched in monocytes (IV) (M118)	51	-1.68	0.004	0.046	HLA-DMB, MYO1F, LGALS3, ARHGEF10L, MGAM, HAL, NACC2, ACSL1, CARD9, PGD, CD68, AMICA1, PID1, PAK1, SLC24A4, TNFSF13B, LRRK2, PADI4, AGPAT9, EMR1, TMEM176B, NAIP, DOCK5, LY96, IL1R2, LTBR, EMILIN2, CXCR2, SMARCD3, RNASE6, DYSF, ST3GAL6, MOSC1, GPR109B, DOK3, STEAP4, ACPP, FGD4, F5
