# Supplementary material for: Transcriptional blood signatures for active and amphotericin B treated visceral leishmaniasis in India
Source: PLoS Negl Trop Dis. 2019 Aug 16;13(8):e0007673. doi: 10.1371/journal.pntd.0007673 (PMC6713396; doi:10.1371/journal.pntd.0007673)
Supplement: S4 Table — The table shows Enrichr results for analysis of 391 genes concordant for differential expression across two experiments comparing active VL cases with healthy controls. (PDF) [file pntd.0007673.s006.pdf]

S4 Table. Results of gene set enrichment analysis in Enrichr* using 391 genes represented by 439 probes on the arrays which were concordant for differential expression (adjusted P-value <0.05; ≥2-fold change) between active cases and healthy controls in experiments 1 and 2. Only results where the Z score is <-1 or >1, and the enrichment adjusted P-value is <0.01, are included. Enrichr accesses a collection of diverse gene set libraries. Here we provide results for enrichment of genes in our dataset compared to the KEGG 2016, Wiki 2016, Reactome 2016,NCI-Nature 2016 pathways databases, as well as to the PPI Hub Protein, Human Cyc, Jensen Tissues, MGI Mammalian Phenotype, Human Gene Atlas, and BIOCARTA tables.

Database	Term	P-value	Adjusted P-value	Z-score	Combined Score	Genes
Reactome 2016	Cell Cycle_Homo sapiens_R-HSA-1640170	5.7E-10	1.99E-07	-2.46	52.38	TOP2A;CDCA5;HJURP;NCAPG;CDCA8;MCM10;HMMR;FOXM1;TYMS;CENPA;AURKB;CKS1B;CDC20;CCNB2;PTTG1;EXO1;CHEK1;HIST3H2BB;OIP5;BUB1;PLK4;GINS2;UBE2C;CDC25A;ZWINT;CCNA2;TPX2;TFDP1;TFDP2;CCNE2;POLE2;BIRC5;MCM4;CENPN;KIF2C;MCM2
	Cell Cycle, Mitotic_Homo sapiens_R-HSA-69278	6.7E-10	1.99E-07	-2.47	52.13	TOP2A;CDCA5;NCAPG;CDCA8;MCM10;HMMR;FOXM1;TYMS;CENPA;AURKB;CKS1B;CDC20;CCNB2;PTTG1;HIST3H2BB;BUB1;PLK4;GINS2;UBE2C;CDC25A;ZWINT;CCNA2;TPX2;TFDP1;TFDP2;CCNE2;POLE2;BIRC5;MCM4;CENPN;KIF2C;MCM2
	Metallothioneins bind metals_Homo sapiens_R-HSA-5661231	1.2E-06	1.46E-04	-1.77	24.19	MT2A;MT1A;MT1G;MT1H;MT1E
	Response to metal ions_Homo sapiens_R-HSA-5660526	1.2E-06	1.46E-04	-1.72	23.56	MT2A;MT1A;MT1G;MT1H;MT1E
	Mitotic Prometaphase_Homo sapiens_R-HSA-68877	1.2E-06	1.46E-04	-2.02	27.43	CDC20;CCNB2;CDCA5;NCAPG;BIRC5;CDCA8;KIF2C;CENPN;CENPA;BUB1;AURKB;ZWINT
	Resolution of Sister Chromatid Cohesion_Homo sapiens_R-HSA-2500257	3.7E-06	3.70E-04	-2.05	25.58	CDC20;CCNB2;CDCA5;BIRC5;CDCA8;KIF2C;CENPN;CENPA;BUB1;AURKB;ZWINT
	G0 and Early G1_Homo sapiens_R-HSA-1538133	6.9E-06	5.88E-04	-1.96	23.25	CCNA2;TOP2A;TFDP1;TFDP2;CCNE2;CDC25A
	Mitotic G1-G1/S phases_Homo sapiens_R-HSA-453279	1.5E-05	1.12E-03	-2.07	23.01	CCNA2;TOP2A;TFDP1;TFDP2;CCNE2;POLE2;MCM4;MCM10;TYMS;CDC25A;MCM2;CKS1B
	G1/S Transition_Homo sapiens_R-HSA-69206	7E-05	4.61E-03	-2.07	19.85	CCNA2;TFDP1;CCNE2;POLE2;MCM4;MCM10;TYMS;CDC25A;MCM2;CKS1B
	Separation of Sister Chromatids_Homo sapiens_ R-HSA-2467813	8.51E-05	5.04E-03	-2.24	21.02	CDC20;PTTG1;UBE2C;CDCA5;BIRC5;CDCA8;KIF2C;CENPN;CENPA;BUB1;AURKB;ZWINT
	Mitotic Anaphase_Homo sapiens_ R-HSA-68882	1.59E-04	8.28E-03	-2.26	19.79	CDC20;PTTG1;UBE2C;CDCA5;BIRC5;CDCA8;KIF2C;CENPN;CENPA;BUB1;AURKB;ZWINT
	Mitotic Metaphase and Anaphase_Homo sapiens_R-HSA-2555396	1.68E-04	8.28E-03	-2.26	19.61	CDC20;PTTG1;UBE2C;CDCA5;BIRC5;CDCA8;KIF2C;CENPN;CENPA;BUB1;AURKB;ZWINT
Wiki Pathways 2016	Retinoblastoma (RB) in Cancer_Homo sapiens_WP2446	2.2E-09	4.91E-07	-1.95	38.91	TOP2A;PLK4;TTK;TYMS;CDC25A;CCNA2;CCNB2;TFDP1;TFDP2;CCNE2;POLE2;CHEK1;STMN1;MCM4
	Cell Cycle_Homo sapiens_WP179	1.1E-07	1.21E-05	-1.87	29.95	MCM10;CDC25A;CDC20;CCNA2;CCNB2;TFDP1;PTTG1;CCNE2;CHEK1;MCM4;BUB1;PTTG3P;MCM2
KEGG 2016	Cell cycle_Homo sapiens_hsa04110	9.7E-07	1.79E-04	-1.73	24.01	TTK;CDC25A;CDC20;CCNA2;CCNB2;TFDP1;PTTG1;TFDP2;CCNE2;CHEK1;MCM4;BUB1;MCM2
NCI_Nature 2016	Aurora B signaling_Homo sapiens_304a75af-618c-11e5-8ac5-06603eb7f303	7.19E-07	4.15E-05	-1.39	19.70	STMN1;BIRC5;CDCA8;NCAPG;KIF2C;CENPA;BUB1;AURKB
	FOXM1 transcription factor network_Homo sapiens_c51cda49-6192-11e5-8ac5-06603eb7f303	8.84E-07	4.15E-05	-1.65	23.02	CCNA2;CCNB2;BIRC5;TGFA;FOXM1;CENPA;AURKB;CKS1B
	Aurora A signaling_Homo sapiens_f131cf8e-618b-11e5- 8ac5-06603eb7f303	3.11E-04	9.75E-03	-1.46	11.77	TPX2;BIRC5;CENPA;AURKB;DLGAP5
PPI Hub Proteins Table	CDK1	2.9E-07	8.14E-05	-2.15	32.40	TOP2A;ARF4;EPB41;NCAPG;KIF11;FOXM1;AURKB;RAP1GAP;CKS1B;CDC20;CCNB2;PTTG1;EXO1;CA2;CHEK1;STMN1;PBK;PHGDH;TK1;BUB1;CEP55;DLGAP5;TLE3;SVIL;DUSP1;ANK1;CDC25A;CCNA2;TPX2;RARA;BIRC5;MCM4;KIF2C;CDKN3
Jensen Tissues Table	Erythroid_cell	4.7E-06	0.002	-6.78	83.14	HJURP;CDC20;CHEK1;NUSAP1;OIP5;HIST3H2BB;TGM2;TNS1;TMOD1;BNIP3L;TPM1;KRT1;ACSL6;ANK1;CDC25A;KEL;MELK;HMBS;LPPR2;TRIM10;RHCE;OSBP2;TSTA3;EPB42;FECH;TROAP;CDCA5;GYPE;CDCA8;GMPR;NCAPG;HMMR;SLC1A5;EPB49;SPTA1;CCNB2;PRDX2;PLEK2;SLC15A4;SLC38A5;PLK4;SLC14A1;UBE2C;SIAH2;HPS1;TNFRSF10B;CYP4F12;ZWINT;TPX2;SLC6A8;SLC6A9;UBE2T;UBE2O;TMCC2;BCL2L1;RSPH10B;TOP2A;ARF4;RNF10;ALAS2;DYRK3;RNF14;FHL2;MCM10;MYL6B;HBD;KIF11;KIF15;CKS1B;MMP25;CA1;CA2;PBK;TK1;ACP1;DLGAP5;DUSP1;RAB3IL1;BPGM;SPTB;EPN2;ETV7;ASPM;SLC7A5;TFDP1;TFDP2;TRIM58;MCM4;BIRC5;KIF2C;SLC29A1;SGK1;ST6GALNAC4;MCM2;HEMGN;KANK2;TTK;TYMS;AURKB;RHD;SELENBP1;CREG1;MXI1;AHSP;IGF2BP2;BUB1;CTNNAL1;SNCA;RBM38;GINS2;SMOX;FAM46C;RIOK3;MBOAT7;AKR1C3;KLF1;XK;KIAA0101;POLE2;TMEM56;CENPN;CHPT1;RHAG;TRIP13;CDKN3

MGI Mammalian Phenotype 2017	MP:0002447_abnormal_ erythrocyte_morphology	5.3E-10	8.07E-07	-2.91	62.26	TMOD1;RBM38;BNIP3L;FECH;EPB42;EPB41;SPTB;ANK1; KLF1;SPTA1;PRDX2;MCM4;AHSP;MCM2
	MP:0000245_abnormal_ erythropoiesis	2.2E-08	1.7E-05	-2.57	45.28	DYRK3;SPI1;ST6GALNAC2;FECH;EPB41;ANK1;CDC25A; KLF1;SPTA1;PRDX2;IFNG;HLX;AHSP;SLC19A1
	MP:0003656_abnormal_ erythrocyte_physiology	9.5E-08	3.65E-05	-2.29	37.09	SPTA1;BNIP3L;AQP9;RHAG;ANK1;SPTB;RHCE
	MP:0002812_spherocytosis	8.2E-08	3.65E-05	-2.20	35.91	BNIP3L;PRDX2;EPB42;EPB41;ANK1;SPTB
	MP:0000208_decreased_ hematocrit	2.4E-07	7.42E-05	-2.85	43.38	RBM38;BNIP3L;SPI1;EPB42;FECH;EPB41;FBLN1;ANK1; SPTB;KLF1;SPTA1;PRDX2;SOCS1;AHSP;TGM2
	MP:0002640_reticulocytosis	1.1E-05	2.80E-03	-2.60	29.75	SPTA1;BNIP3L;PRDX2;EPB42;EPB41;MCM4;AHSP;SPTB; ANK1
	MP:0003657_abnormal_ erythrocyte_osmotic_lysis	1.4E-05	0.003	-2.16	24.14	SPTA1;BNIP3L;AQP9;ANK1;SPTB;KLF1
	MP:0001577_anemia	2.1E-05	0.004	-3.11	33.50	TMOD1;RBM38;BNIP3L;ALAS2;SPI1;EPB42;ALOX15;ANK1;S PTB;KLF1;SPTA1;IFNG;HLX;AHSP;BUB1
	MP:0004046_abnormal_mitosis	1.8E-05	0.004	-2.47	26.94	CDC20;PLK4;NUSAP1;CDCA8;CENPA;BUB1;CDC25A; AURKB
	MP:0005639_hemosiderosis	2.8E-05	0.004	-1.66	17.46	SPTA1;PRDX2;TK1;ANK1
	MP:0002591_decreased_mean_ corpuscular_volume	6.1E-05	0.009	-2.75	26.67	SPTA1;BNIP3L;RNF10;EPB42;EPB41;FAM46C;AHSP;ANK1;R HCE
Human Gene Atlas	CD71+_EarlyErythroid	1.6E-38	1.2E-36	-1.96	170.88	RNF10;ALAS2;RNF14;HBD;FOXM1;KIF15;CDC20;CA1; PTTG1;CA2;NUSAP1;OIP5;ACP1;DLGAP5;TNS1;TMOD1; BNIP3L;ACSL6;BPGM;ANK1;SPTB;CDC25A;EPN2;MYL4; CCNA2;ASPM;KEL;TFDP1;CCNE2;TFDP2;KIFC1;HMBS; TRIM58;BIRC5;SLC29A1;ST6GALNAC4;TRIM10;RHCE; OSBP2;HEMGN;KANK2;NFIX;FECH;EPB42;GYPB;EPB41; GYPE;NCAPG;GMPR;TTK;HMMR;EPB49;RHD;SPTA1; SELENBP1;PRDX2;C20ORF108;SEC14L4;MXI1;AHSP;PLEK2;S LC38A5;SNCA;RBM38;PLK4;SLC14A1;RIOK3;SMOX; SIAH2;HPS1;KLF1;DARC;XK;SLC6A8;SLC6A9;TMEM56; UBE2T;UBE2O;RHAG;CHPT1;TMCC2;CENPN;HBBP1; PTTG3P;BCL2L1
Jensen Diseases	Congenital_hemolytic_anemia	8E-08	3.46E-05	-2.89	47.29	SPTA1;EPB42;EPB41;RHAG;BPGM;ANK1;SPTB;KLF1
	Hereditary_elliptocytosis	2.1E-06	4.53E-04	-3.91	51.08	SPTA1;EPB41;ANK1;SPTB
	Kernicterus	4.3E-05	6.15E-03	-3.02	30.42	HAL;EPB42;RHCE;RHD
BioCarta 2016	Hemoglobin's Chaperone_Homo sapiens_h_ahspPathway	8.9E-05	0.005	-1.32	12.30	ALAS2;FECH;HMBS;AHSP

* Chen, E.Y. et al. Enrichr: interactive and collaborative HTML5 gene list enrichment analysis tool. BMC Bioinformatics 14, 128 (2013).
Kuleshov, M.V. et al. Enrichr: a comprehensive gene set enrichment analysis web server 2016 update. Nucleic Acids Res 44, W90-7 (2016).
