# Supplementary material for: Transcriptional blood signatures for active and amphotericin B treated visceral leishmaniasis in India
Source: PLoS Negl Trop Dis. 2019 Aug 16;13(8):e0007673. doi: 10.1371/journal.pntd.0007673 (PMC6713396; doi:10.1371/journal.pntd.0007673)
Supplement: S5 Table — The table shows Enrichr results for analysis of 210 genes concordant for differential expression across two experiments comparing active VL cases with treated VL cases. (PDF) [file pntd.0007673.s007.pdf]

S5 Table. Results of gene set enrichment analysis in Enrichr* using 210 genes represented by 221 probes on the arrays which were concordant for differential expression (adjusted P-value <0.05) between active cases and cured cases in experiments 1 and 2. Only results where the Z score is <-1 or >1, and the enrichment adjusted P-value is ≤.05, are included. Enrichr accesses a collection of diverse gene set libraries. Here we provide results for enrichment of genes in our dataset compared to the Reactome 2016, Wiki 2016, KEGG 2016,NCI-Nature 2016 pathways databases, as well as to the Human Gene Atlas Table and the LINCS_L1000_ligand_perturbations_up Table1.

Database	Term	P-value	Adjusted P-value	Z-score	Combined Score	Genes
Reactome 2016	Chemokine receptors bind chemokines_Homo sapiens_ R-HSA-380108	3.01E-04	0.05	-1.97	15.94	CCL25;CXCL10;CXCL11;CXCL9;CXCR1
	NICD traffics to nucleus_Homo sapiens_R-HSA-157052	3.02E-04	0.05	-2.19	17.78	NOTCH1;MAML3;RBPJ
	Notch-HLH transcription pathway_Homo sapiens_ R-HSA-350054	3.02E-04	0.05	-2.15	17.44	NOTCH1;MAML3;RBPJ
	Pre-NOTCH Expression and Processing_Homo sapiens_ R-HSA-1912422	3.27E-04	0.05	-2.22	17.80	LFNG;NOTCH1;TFDP2;MAML3;RBPJ
Wiki Pathways 2016	Type II interferon signaling (IFNG)_Homo sapiens_WP619	2.2E-06	4.69E-04	-1.91	24.84	CXCL10;CXCL9;SPI1;IFNG;STAT1;GBP1
	Type II interferon signaling (IFNG)_Mus musculus_WP1253	3.37E-04	0.026	-1.81	14.49	CXCL10;SPI1;IFNG;STAT1
	Chemokine signaling pathway_Mus musculus_WP2292	3.63E-04	0.026	-2.08	16.47	LYN;CCL25;CXCL10;NRAS;CXCR1;STAT1;PLCB2;MAPK3
	IL-3 Signaling Pathway_Mus musculus_WP373	5.57E-04	0.030	-2.02	15.10	LYN;RXRA;SPI1;STAT1;RARA;MAPK3
	Notch Signaling Pathway_Homo sapiens_WP268	1.36E-03	0.048	-1.94	12.77	LFNG;NOTCH1;MAML3;RBPJ
	Notch Signaling Pathway_Mus musculus_WP29	1.36E-03	0.048	-1.85	12.18	LFNG;NOTCH1;MAML3;RBPJ
	Delta-Notch Signaling Pathway_Mus musculus_WP265	1.63E-03	0.049	-1.81	11.63	LFNG;NOTCH1;MAML3;RBPJ;MAPK3
KEGG 2016	Chemokine signaling pathway_Homo sapiens_hsa04062	3E-05	5.79E-03	-1.93	20.11	LYN;CCL25;CXCL10;CXCL11;NRAS;CXCL9;CXCR1;STAT1; PLCB2;MAPK3
	Pathways in cancer_Homo sapiens_hsa05200	8.3E-05	8.14E-03	-2.08	19.57	CSF1R;SPI1;CSF3R;STAT1;LPAR2;RASGRP4;CKS1B;NRAS; RXRA;CCNE2;RARA;CKS2;PLCB2;MAPK3
	Viral carcinogenesis_Homo sapiens_hsa05203	3.28E-04	0.021	-1.93	15.50	LYN;NRAS;GSN;HIST1H4K;CCNE2;ACTN1;TBPL1;RBPJ; MAPK3
	Osteoclast differentiation_Homo sapiens_hsa04380	4.96E-04	0.024	-1.79	13.60	CSF1R;SPI1;IFNG;STAT1;NCF4;LILRB3;MAPK3
	Phospholipase D signaling pathway_Homo sapiens_hsa04072	8.31E-04	0.033	-1.79	12.73	NRAS;CYTH4;CXCR1;LPAR2;PIP5K1C;PLCB2;MAPK3
	Thyroid hormone signaling pathway_Homo sapiens_hsa04919	1.55E-03	0.045	-1.73	11.18	NRAS;RXRA;NOTCH1;STAT1;PLCB2;MAPK3
	Notch signaling pathway_Homo sapiens_hsa04330	1.60E-03	0.045	-1.50	9.65	LFNG;NOTCH1;MAML3;RBPJ
NCI_Nature 2016	EGF receptor (ErbB1) signaling pathway_Homo sapiens_NULL	2.6E-05	3.49E-03	-1.81	19.03	NRAS;GSN;STAT1;PIP5K1C;MAPK3
	CXCR3-mediated signaling events_Homo sapiens_3a38a0ca-6191-11e5-8ac5-06603eb7f303	8.5E-05	5.59E-03	-1.71	16.01	CXCL10;CXCL11;CXCL9;NRAS;MAPK3
	GMCSF-mediated signaling events_Homo sapiens_095aa3ef-6193-11e5-8ac5-06603eb7f303	5.35E-04	0.024	-1.54	11.59	LYN;NRAS;STAT1;MAPK3
Human Gene Atlas	CD14+_Monocytes	7.3E-10	2.3E-08	-1.97	41.46	CSF1R;SLC27A1;SPI1;CTDP1;LST1;RASGRP4;PPM1F;RXRA; ALDH3B1;ITGAX;CHST15;SLC15A3;SLC12A9;MAP3K3;CD93; DNAJB12;CACNA2D3;MYO9B;LILRB3;DUSP6;SLCO3A1; TBXAS1;PECAM1;DPEP2;TKT;PLCB2;SIGLEC7;GAS7
	CD33+_Myeloid	3.09E-04	0.006	-2.21	17.90	CSF1R;NDUFB3;PTAFR;LPAR2;RASGRP4;PPM1F;RXRA; RAB11FIP1;ALDH3B1;CD300LB;CHST15;AMICA1;SLC15A3; KDM6B;MAP3K3;RNF24;CD93;STK11IP;RNASET2;ALG13; FLJ10357;MYO9B;LILRB3;DUSP6;TBXAS1;PECAM1;TKT; PLCB2;SIGLEC7;GAS7
LINCS_L1000_ligand _ perturbations_up Table 1	TNFA-HS578T	1.7E-07	1.61E-05	-1.90	29.68	CXCL10;CXCL11;CXCL9;SERPINA1;GCH1;STAT1;NAMPT; FAM129A;PHLDA1;GBP1;FBP1;DUSP6
	IFNG-HS578T	2E-06	9.6E-05	-1.45	19.01	CXCL10;CXCL11;CXCL9;GSN;GCH1;STAT1;NAMPT;GBP1; FBP1;DUSP6
	IL1-BT20	2.4E-05	6.37E-04	-1.52	16.16	LYN;CXCL10;CXCL11;SERPINA1;GCH1;NAMPT;PHLDA1; GBP1;DUSP6
	IL1-HS578T	2.7E-05	6.37E-04	-1.51	15.88	CXCL10;SERPINA1;GCH1;STAT1;NAMPT;PI3;PHLDA1; GBP1;DUSP6
	TNFA-BT20	3.4E-05	6.37E-04	-1.56	16.04	LYN;CXCL10;CXCL11;GCH1;NAMPT;PI3;PHLDA1;GBP1; DUSP6
	IFNA-BT20	0.0001	1.61E-03	-1.17	10.71	CXCL10;CXCL11;CXCL9;GCH1;STAT1;CD38;SLC15A3;GBP1
	IL6-SKBR3	1.44E-04	1.95E-03	-1.11	9.79	NOTCH1;SERPINA1;CD93;NAMPT;CHI3L1;TGFBI;PHLDA1;G BP1
	TNFA-MCF7	2.83E-04	3.36E-03	-1.55	12.65	CXCL10;RAB11FIP1;SERPINA1;GCH1;SLCO3A1;NAMPT; FAM129A;GBP1
	IFNG-MDAMB231	4.73E-04	5.00E-03	-0.86	6.61	CXCL10;CXCL11;CXCL9;GCH1;STAT1;NAMPT;GBP1
	IFNG-BT20	5.92E-04	5.58E-03	-0.87	6.45	CXCL11;CXCL9;GCH1;STAT1;CD38;GBP1;DUSP6
	IFNA-SKBR3	6.46E-04	5.58E-03	-0.90	6.60	CXCL10;CXCL11;CXCL9;STAT1;NAMPT;TGFBI;GBP1
	IL1-MCF10A	9.01E-04	7.14E-03	-0.86	6.01	CXCL10;CXCL11;GCH1;FAM129A;MT1G;CHI3L1;GBP1
	IFNA-HS578T	1.02E-03	7.42E-03	-1.00	6.88	CXCL10;CXCL11;STAT1;CD38;PHLDA1;GBP1;DUSP6
	HGF-MCF7	1.48E-03	9.10E-03	-1.17	7.65	CXCL10;CXCL11;CHST15;CHI3L1;CD38;PI3;DUSP6
	EGF-MCF10A	1.53E-03	9.10E-03	-1.16	7.54	CKS2;FAM64A;PI3;HMMR;CDC25C;PHLDA1;DUSP6
	HGF-BT20	1.53E-03	9.10E-03	-1.06	6.88	CXCL10;CXCL11;CXCL9;SERPINA1;ALG13;PHLDA1;DUSP6

* Chen, E.Y. et al. Enrichr: interactive and collaborative HTML5 gene list enrichment analysis tool. BMC Bioinformatics 14, 128 (2013).
Kuleshov, M.V. et al. Enrichr: a comprehensive gene set enrichment analysis web server 2016 update. Nucleic Acids Res 44, W90-7 (2016).
