# Supplementary material for: Transcriptional blood signatures for active and amphotericin B treated visceral leishmaniasis in India
Source: PLoS Negl Trop Dis. 2019 Aug 16;13(8):e0007673. doi: 10.1371/journal.pntd.0007673 (PMC6713396; doi:10.1371/journal.pntd.0007673)
Supplement: S6 Table — The table shows Enrichr results for analysis of 988 genes differentially expressed between active VL cases and treated VL cases in experiment 2 but not experiment 1. (PDF) [file pntd.0007673.s008.pdf]

S6 Table. Results of gene set enrichment analysis in Enrichr* using 988 genes represented by 1096 probes on the arrays which were significant for differential expression (adjusted P-value <0.05) between active cases and cured cases in experiments 2 but not in experiment 1. Only results where the Z score is <-1 or >1, and the enrichment adjusted P-value is ≤.05, are included. Enrichr accesses a collection of diverse gene set libraries. Here we provide results for enrichment of genes in our dataset compared to the Reactome 2016, Wiki 2016, KEGG 2016,NCI-Nature 2016 pathways databases, as well as to the LINCS_L1000_ligand_perturbations_down Table.

Database	Term	P-value	Adjusted P-value	Z-score	Combined Score	Genes
Reactome 2016	Cell Cycle, Mitotic_Homo sapiens_R-HSA-69278**	3.5E-34	3.95E-31	-2.48	190.81	TOP2A;AHCTF1;FEN1;ERCC6L;MCM8;ZWILCH;GMNN;CASC5;BUB1B;MCM10;FOXM1;SMC2;CKS1B;CDC20;SGOL2;SGOL1;PTTG1;PPP2R1B;NUF2;NEK2;FBXO5;NDEL1;NUP214;RFC5;RFC4;HIST1H2AJ;VRK1;CSNK1D;KIF23;ESCO2;CDC25C;TUBG1;CDC25A;CCNA2;PSMA3;PSMA4;PSMA1;ESPL1;CCNE1;BIRC5;MCM4;KIF2C;MCM6;KIF20A;MCM2;PCNA;PSMD14;CDCA5;PRIM1;CUL1;NCAPG;CDCA8;HMMR;PKMYT1;TYMS;CENPA;PMF1;AURKB;AURKA;PSMB6;CCNB2;CCNB1;PSMB2;BUB1;MAPK3;PLK4;GINS2;RRM2;CDKN2A;UBE2C;NEK6;GINS3;PLK1;H3F3A;CDC7;NDC80;ZWINT;CENPE;TPX2;CENPF;KIF18A;APITD1;RPS27;CENPH;CENPI;RPA3;POLE2;PSMC2;CDK2;CENPL;CENPM;CENPN;CENPO;MAD2L1;SPC25;NUP37
	Interleukin-3, 5 and GM-CSF signaling_Homo sapiens_R-HSA-512988	0.003	0.032	-1.71	10.15	STAT5B;PSMD14;DUSP1;IL5RA;PIK3CD;IRS2;ARRB1;GAB2;ARRB2;CBL;GFRA2;CSF2RA;RASGRP4;PSMB6;PSMA3;PSMA4;PSMA1;PSMB2;PPP2R1B;PSMC2;RAF1;CAMK2G;HBEGF;MAPK3
	Interleukin-2 signaling_Homo sapiens_R-HSA-451927	0.003	0.041	-1.66	9.36	STAT5B;PSMD14;DUSP1;IL5RA;PIK3CD;IRS2;ARRB1;GAB2;ARRB2;GFRA2;CSF2RA;RASGRP4;PSMB6;PSMA3;PSMA4;PSMA1;PSMB2;PPP2R1B;PSMC2;RAF1;CAMK2G;HBEGF;MAPK3
Wiki Pathways 2016	Cell Cycle_Homo sapiens_WP179**	2.3E-11	3.89E-09	-1.87	45.76	PCNA;MCM8;BUB1B;MCM10;PKMYT1;CDC20;CCNB2;CCNB1;PTTG1;CHEK1;BUB1;CDKN2A;PLK1;CDC7;CDC25C;CDC25A;CCNA2;ESPL1;CCNE1;CDK2;MCM4;MCM6;PTTG3P;MAD2L1;MCM2
	IL-1 Signaling Pathway_Mus musculus_WP37	3.03E-04	0.007	-1.53	12.43	CAPNS1;IL1R1;IL1R2;AKT1;SIRPA;IL1RAP;PRKCZ;MAPK3
	IL-3 Signaling Pathway_Homo sapiens_WP286	5.83E-04	0.013	-1.78	13.25	STAT5B;AKT1;IL5RA;PIK3CD;GAB2;RAF1;CBL;CCR3; MAPK3
	IL-7 Signaling Pathway_Mus musculus_WP297	0.001	0.018	-1.36	9.30	CCNA2;STAT5B;CDK2;AKT1;IRS2;RAF1;CBL;MAPK3
	IL-4 Signaling Pathway_Homo sapiens_WP395	0.001	0.019	-1.50	10.10	STAT5B;FES;AKT1;BIRC5;PIK3CD;IRS2;GAB2;CBL;MAPK3
	Apoptosis Modulation and Signaling_Homo sapiens_WP1772	0.002	0.028	-1.43	8.84	HRK;CASP7;CAPNS1;IL1R1;CDKN2A;IL1R2;TNFRSF10B;BIRC5;FASLG;BMF;TNFRSF1A;MAPK3
	Delta-Notch Signaling Pathway_Mus musculus_WP265	0.002	0.028	-1.32	8.14	NOTCH2;LFNG;APH1B;HEY1;CDK2;CUL1;AKT1;HES1;WDR12;SAP30;MAPK3
	IL-3 Signaling Pathway_Mus musculus_WP373	0.003	0.036	-1.41	8.20	STAT5B;PAK1;SPI1;FES;CHEK1;AKT1;BIRC5;PIK3CD;GAB2;RAF1;CBL;MAPK3
	IL-6 signaling Pathway_Mus musculus_WP387	0.003	0.036	-1.37	7.99	NCOA1;STAT5B;PPP2R1B;FES;RPS6KA2;PRKCD;BTK;AKT1;GAB2;RAF1;SGK1;MAPK3
	Chemokine signaling pathway_Mus musculus_WP2292	0.003	0.038	-1.37	7.81	CCL25;STAT5B;PRKCD;PIK3CD;ARRB1;CXCR6;ARRB2;PRKCZ;CXCL16;GNAI2;TIAM2;PAK1;GNG7;AKT1;RAF1;CCR3;MAPK3
KEGG 2016	Cell cycle_Homo sapiens_hsa04110**	1.6E-09	4.25E-07	-1.73	35.13	CDKN1C;PCNA;CUL1;BUB1B;TTK;PKMYT1;CDC20;CCNB2;CCNB1;PTTG1;CHEK1;BUB1;CDKN2A;PLK1;CDC7;CDC25C;CDC25A;CCNA2;ESPL1;CCNE1;CDK2;MCM4;MCM6;MAD2L1;MCM2
	Chemokine signaling pathway_Homo sapiens_hsa04062	0.002	0.050	-1.67	10.11	CCL25;STAT5B;CCL23;PRKCD;PIK3CD;ARRB1;CXCR6;ARRB2;PRKCZ;CXCL5;CXCL16;GNAI2;PAK1;GNG7;CCL3;AKT1;RAF1;CCR3;MAPK3
	Fc gamma R-mediated phagocytosis_Homo sapiens_hsa04666	0.002	0.050	-1.63	10.10	VASP;PAK1;MARCKSL1;FCGR2A;PRKCD;ARPC5L;AKT1;PIK3CD;ASAP1;GAB2;RAF1;MAPK3
NCI_Nature 2016	Aurora B signaling_Homo sapiens_304a75af-618c-11e5-8ac5-06603eb7f303**	1.4E-11	2.26E-09	-1.39	34.76	CDCA8;NCAPG;KIF23;CENPA;NDC80;AURKB;AURKA;SMC2;PSMA3;SGOL1;RACGAP1;STMN1;BIRC5;KIF2C;KIF20A;BUB1
	IL8- and CXCR2-mediated signaling events_Homo sapiens_fe78e284-6193-11e5-8ac5-06603eb7f303	1.98E-04	0.005	-1.23	10.50	VASP;GNA15;AKT1;ARRB1;ARRB2;CBL;PIK3R6;GNAI2
	IL4-mediated signaling events_Homo sapiens_cff33f50-6193-11e5-8ac5-06603eb7f303	6.57E-04	0.012	-1.28	9.34	IL10;STAT5B;SPI1;FES;SP1;ALOX15;AKT1;IRS2;CBL; IL13RA1
	CXCR4-mediated signaling events_Homo sapiens_46a5529b-6191-11e5-8ac5-06603eb7f303	0.004	0.047	-1.10	6.13	STAT5B;PAK1;RGS1;AKT1;PIK3CD;ITGA5;ARRB2;RHOC;PRKCZ;PIK3R6;RHOB;GNAI2
LINCS_L1000_ligand_ perturbations_ down	IL4-HS578T	6.6E-13	6.36E-11	-1.81	50.84	TOP2A;GMNN;KRT23;TTK;KIF11;NDRG2;F11R;MKI67 CENPA;AURKB;CKS1B;RAD51AP1;ALOX5;PBK;NUSAP1;BUB1;CEP55;E2F8;FANCI;RFC5;CD163;RRM2;UBE2C;ZWINT;CCNA2;CENPE;ASPM;CENPF;DHRS7;MELK;KIAA0101;BIRC5;DTL;MAD2L1
	MSP-SKBR3	4.51E-04	0.022	-1.78	13.73	CFD;RFC5;TOP2A;ACOT7;RRM2;TNFSF13;HMMR;TYMS;CKS1B;CCNA2;OLFM1;SLPI;PRC1;ANPEP;RRAGD;PLEKHM1;NUSAP1;CD302;ZNF467
	TGFA-BT20	0.001	0.033	-1.48	10.16	CFD;RTN3;CD163;PCNA;CHKA;PRKCD;FBLN1;TYMS;NDC80;CXCL5;CDC20;CST3;GNA15;RAB11FIP1;NPEPL1;FCER1A;CFB

* Chen, E.Y. et al. Enrichr: interactive and collaborative HTML5 gene list enrichment analysis tool. BMC Bioinformatics 14, 128 (2013).
Kuleshov, M.V. et al. Enrichr: a comprehensive gene set enrichment analysis web server 2016 update. Nucleic Acids Res 44, W90-7 (2016). **Only the top cell cycle-related pathways are shown; many more significant cell cycle-related pathways not shown.
