# Supplementary material for: Transcriptional blood signatures for active and amphotericin B treated visceral leishmaniasis in India
Source: PLoS Negl Trop Dis. 2019 Aug 16;13(8):e0007673. doi: 10.1371/journal.pntd.0007673 (PMC6713396; doi:10.1371/journal.pntd.0007673)
Supplement: S7 Table — The table shows Enrichr results for 417 genes differentially expressed between active VL cases and treated VL cases in experiment 1 but not experiment 2, with additional gene sets (see also S4 Table) enriched between active VL cases and treated VL cases in experiment 2 but not experiment 1. (PDF) [file pntd.0007673.s009.pdf]

S7 Table. Results of gene set enrichment analysis in Enrichr* using 417 genes (from 443 probes) which were significant for differential expression (adjusted P-value <0.05) between active cases and cured cases in experiment 1 **but not** experiment 2. Comparative results are shown for the same pathways using 988 genes (from 1096 probes) which were significant for differential expression (adjusted P-value <0.05) between active cases and cured cases in experiments 2 **but not** in experiment 1. Only results where the Z score is <-1 or >1, and the enrichment adjusted P-value is ≤0.05, are included. Enrichr accesses a collection of diverse gene set libraries. Here we provide results for enrichment of genes in our dataset compared to the Human Gene Atlas and the PPI Hub Proteins databases.

Database	Term	P-value	Adjusted P-value	Z-score	Combined Score	Genes
**Enrichr results for genes differentially expressed between active cases and cured cases in experiment 1 but not experiment 2**						
Human Gene Atlas Table	CD71+_EarlyErythroid	0.0003	0.016	-1.96	16.17	ISCA1;CMAS;ABCB7;GTF2B;SFRS2B;TRAK2;IFT52;OPA1;PDCD10;PDDC1;ACP1;IL15RA;PCGF5;MAD2L1BP;BPGM; NTAN1;TUBG1;DCK;ELL2;RBX1;CUL4A;KAT2B;PPM1A;GCLC;ABCB10;TFDP2;TMEM11;EHBP1L1;WDR51B;DHX29;HBBP1
SILAC Phosphoproteomics Table	down 60min_BMP4 vs ctrl_hESC (Human) [19664995]	5.4E-05	0.003	-1.74	17.13	ATF2;SMARCD2;BAT2;NUFIP2;STAU2;CHD4;TCF20;BICD2;ATXN2;ZMYM2;POLR2A;TRIM24;PAK2;RBM10;AZI1;SRRM2;CDC2L2;ANKHD1-EIF4EBP3;CD2AP;NCOR2;EIF3J;TACC3;CCNYL1;TERF2IP;DHX29;VIM;LRCH4
PPI Hub Proteins	NFKBIA	1.17E-04	0.009	-1.81	16.35	NCOR2;RWDD3;HOXA9;TBK1;UBE2D2;UBE2E3;COPS2; UBE2D3;SKP1A;JAK2;ZNF212
	GSK3B	3.44E-04	0.015	-1.79	14.25	C14ORF129;BAT2;ATN1;TCF20;ARID4B;CHD4;KRBA1;BICD2;ZMYM2;BCL2L11;SFRS5;POLR2A;SNRNP70;ADRBK1;SAFB2;RREB1;ANKRD6;SRRM2;CDC2L2;UBE2E3;UPF3A;SORBS3;NCOR2;NFIC;TACC3;TERF2IP;ARHGEF2;RBMX; MAP3K11
	COPS6	2.66E-04	0.013	-1.72	14.16	CUL4A;ZYG11B;MYCBP;CASP8;COPS2;PSAP;DLEU1; KLHL22;VIM;UCHL3;RBX1
	CREBBP	2.46E-04	0.013	-1.38	11.48	IFNAR2;MYST3;STAT5A;ATF2;CREBBP;GMEB2;GTF2B;NCOR2;KAT2B;CDK8;HOXA9;SOCS1;POLR2A;NFIC;CSK; PCAF;MAP3K5;CUTL1
	YWHAE	6.09E-04	0.023	-1.52	11.28	SRRM2;YWHAB;GTF2B;SAMSN1;DNAJA1;NCOR2;TBK1;HIST2H4B;TFDP2;ARHGEF2;VIM;RBM10;MAP3K5
	SMAD9	1.55E-03	0.047	-1.63	10.54	ARHGAP9;KAT2B;ZNF484;SNRNP70;PSAP;TBCD;UBQLN1;MAN2B1;PHKA2
**Enrichr results for genes differentially expressed between active cases and cured cases in experiment 2 but not experiment 1**						
Human Gene Atlas Table	721_B_lymphoblasts	6E-09	2.38E-07	-2.61	49.32	ZWILCH;DSCC1;GMNN;NCF4;PDCD5;BCCIP;BUB1B;MKI67;GABPB1;SMC2;CDC20;PPP2R1B;SNRPD1;PPAT; CHEK1;NUSAP1;TNFRSF8;OIP5;FBXO5;TIPIN;UTP11L;ESCO2;CTPS;CDC25C;WDR76;CDC25A;SAP30;ARMC1;MELK;MTHFD1;PPA1;MTHFD2;RAB35;FSCN1;TMEM126A;PPIF;SNRPG;KIF20A;PSMD14;CDCA3;CDCA5;TROAP;MRPL18;CDCA8;MRPL17;NCAPG;UBFD1;LIN7A;MRPL14;HMMR;ACAT2;C1ORF112;GTF2E2;RAD51AP1;KIAA1524; CCNB1;RACGAP1;FIGNL1;C13ORF34;C16ORF59;ICOS;NDUFV2;FANCI;FANCL;PLK1;TNFRSF10B;C10ORF33;PAICS;NDC80;ZWINT;MRPL22;COQ5;SQLE;TPX2;UCK2; KIF18A;UBE2T;CDK2;INTS7;FAM54A;EZH2;TOP2A;C2ORF43;FEN1;ORC1L;MCM8;KIF14;MCM10;TRIAP1;KIF11;FOXM1;MRPL35;CKS1B;MRPL3;CHAF1B;PBK;TK1;CEP55;DLGAP5;MRPS28;RFC5;HELLS;CCT2;MAP4K2;PARP3;RFC4;NCBP1;CCDC34;CCNA2;OBFC2B;ASPM; PSMA3;PSMA1;ESPL1;CKS2;BIRC5;MCM4;WDR51A;SNRPA1;KIF2C;MCM6;GART;DTL;MCM2;GTF3C3;GRAMD1A;PCNA;PRIM1;POMP;DENND3;MKI67IP; C14ORF142;CENPA;NAPG;AURKB;HSPD1;AURKA;CAND1;PSMB2;OXCT1;EXOSC8;RAD54L;EXOSC3;BUB1; E2F8;GINS2;RANBP1;PPIL1;RRM1;RRM2;SPAG5;CD70;GINS3;SAAL1;DONSON;ORC6L;MLH1;SHCBP1;BATF;CENPE;CENPF;TTLL4;CENPH; CENPI;PSAT1;PRC1; TOMM70A;EIF3I;CENPM;CENPN;RAN;NUP37
	CD105+_Endothelial	1.8E-07	4.67E-06	-1.96	30.52	TOP2A;FEN1;ORC1L;GMNN;BCCIP;MCM10;MRPL35;C1ORF135;SMC2;PPP2R1B;DLGAP5;LRRCC1;RFC5;MRPS28;WDHD1;RFC4;VRK1;ESCO2;ARMC1;C12ORF48;CCNE1;TIMELESS;CKS2;DNAJC9;SLC29A1;DTL;MCM2;KLHL18;RNASEH2B;TROAP;NCAPG;TTK;UBFD1;AURKA;CCNB2;KIAA1524;RACGAP1;EXOSC8;UBN1;REPS2;EXOSC3;E2F8;FANCI;PLK4;DUT;RRM1;RRM2;ORC6L;CDC7;MLH1;SHCBP1;NDC80;COPS3;CENPF;KIAA0101;PRC1;KIF4A;POLE2;RPA3;CDK2;PSMG1;CENPN;FAM54A;NUP37;METAP2
	CD33+_Myeloid	3.6E-05	0.000578	-2.14	21.90	DOCK5;CDA;IRS2;PYGL;FRY;TREM1;FRAT1;GLT1D1;COL4A3BP;ZXDC;TOM1;IDS;HOMER3;NUDT16;VENTX;IL13RA1;HGSNAT;KLF11;DUSP1;PRKCD;RNASE6;GAB2;ARAP1;F5;TYROBP;NAAA;CD302;TRIB1;SGK1;DNASE1L1;HBEGF;PPP1R15A;NOTCH2;EIF2C4;TTYH3;AHNAK;C2ORF68;NDUFB3;FPR1;ASAP1;DENND3;CFP;LILRA2;CSF2RA;AIF1; RASGRP4;GNAI2;RAB11FIP1;OS9;TMEM127;HLX;TDG;ALOX5;NLRP3;TBL1X;CD14;NADK;LRRC25;SEMA4A;CD163;VDR;TNFSF13;EMR1;CARD9;NAGA;NBEAL2;SOD2;CPPED1;NR4A2;DIAPH1;PTPRE;KLF6;IMPDH1;VNN3;FES;P2RX1;PLEKHM1;EIF2C1;SIGLEC7
	CD14+_Monocytes	3.7E-05	5.78E-04	-1.76	17.95	FAM49A;CDA;SPI1;LST1;ARRB1;SNX11;PYGL;TOM1;SIRPA;GPSM3;IL13RA1;NUP214;GAA;DNTTIP1;DNAJB12;RNASE6;TFEB;ARAP1;PISD;TYROBP;NAAA;ORAI3;BTK;CD302;STK40;DNASE1L1;HBEGF;AHNAK;FPR1;LILRA2;CFP;AIF1;RASGRP4;PAK1;TMEM127;HLX;DHRS7B;ALOX5;CD14;TBL1X;NADK;SULT1A2;LRRC25;CD163;EMR1;TNFSF13;NAGA;CPPED1;FCGR2A;SLCO3A1;IMPDH1;CAMK1;SIGLEC7
PPI Hub Proteins	CDK1**	3.2E-15	1.12E-12	-2.15	71.82	CDKN1C;RERE;TOP2A;ERCC6L;FEN1;ORC1L;BUB1B;IRS2;KIF11;FOXM1;MKI67;CKS1B;CDC20;CHAF1B;PTTG1;PPP2R1B;EXO1;XPO6;CHEK1;STMN1;GRB10;PBK;PHACTR1;TK1;CEP55;DLGAP5;NDEL1;NCOA1;NUP214;TLE3;HMGCS1;DUSP1;IL16;CTPS;CDC25C;MAPK8IP3;CDC25A;NME1;CCNA2;ESPL1;CCNE1;ZNF516;CKS2;BIRC5;MCM4;KIF2C;FAM193A;NOTCH2;CDCA2;PCNA;AHNAK;NCAPG;MKI67IP;PKMYT1;AURKB;AURKA;SLC9A1;GNAI2;CCNB2;MUC1;PAK1;CCNB1;RACGAP1;PRDX1;REPS2;BUB1;SVIL;DUT;RRM2;SPAG5;PHF12;ORC6L;CDC7;PAICS;DIAPH1;NSFL1C;TPX2;ANLN;PRC1;ZYX;PTPN2;EZH2;CDKN3
	PLK1	1.4E-10	2.35E-08	-1.77	40.29	TOP2A;GTF3C3;ERCC6L;BUB1B;PKMYT1;AURKA;PSMB6;PAK1;CCNB1;SGOL1;RACGAP1;PSMB2;C13ORF34;FBXO5;BUB1;CEP55;NUP214;SPAG5;PLK1;KIF23;CDC25C;TUBG1;ASPM;PSMA3;PSMA4;ESPL1;PSMA1;PRC1;BIRC5;RAN;MCM2
	MAPK1	5.4E-08	6.2E-06	-2.00	33.49	TOP2A;CALCOCO1;CDCA5;TTK;ARRB1;HMMR;ARRB2;PRKCZ;SLC9A1;PAK1;CCNB1;RPS6KA5;PTTG1;PPP2R1B;RPS6KA2;ALOX5;STMN1;GRB10;NEK2;CEP55;IER3;MAPK3;NDEL1;NCOA1;NUP214;KLF11;STAT5B;H3F3B;DUSP1;MKL1;PRKCD;IL16;GAB2;CDC25C;TNFRSF1A;NR4A2;CENPE;PTPRE;ESPL1;SP1;CCNE1;CDK2;ERF;ZYX;RAF1;SGK1;METAP2
	CDK2	9.4E-08	8.14E-06	-1.92	31.01	FEN1;ORC1L;BCCIP;IRS2;KIF11;FOXM1;MKI67;CKS1B;CHAF1B;PTTG1;XPO6;CHEK1;STMN1;AKT1;TK1;DLGAP5;NDEL1;NCOA1;TLE3;MKL1;TUBG1;CDC25C;CDC25A;SAP30;CCNA2;PTP4A3;MTHFD1;SLCO4A1;CCNE1;BIRC5;MCM4;TAGLN2;SGK1;DTL;MCM2;NOTCH2;PCNA;AHNAK;CUL1;RPS27L;SLC9A1;CCNB2;PAK1;CCNB1;CAMK2G;MAPK3;STAT5B;DUT;MARCKSL1;GSTM2;RRM2;PYCR1;CDC7;PAICS;CENPE;TPX2;CENPF;RPS27;FABP5;SP1;PRC1;CDK2;ZYX;PTPN2;EZH2;CDKN3
	PPP2CA	1.4E-05	7.22E-04	-1.63	18.31	CCT2;STAT5B;VDR;DUSP1;PRKCD;KIF23;WDR12;F11R;IL8RB;PRKCZ;STRN4;RHOB;MRPS9;PAK1;SGOL2;SGOL1;RACGAP1;PPP2R1B;CDK2;AKT1;TRIP13;SGK1;MAPK3;IER3
	MAPK3	1.4E-05	7.22E-04	-1.80	20.14	TOP2A;TTYH3;CDCA5;TTK;ARRB1;HMMR;ARRB2;PRKCZ;RPS6KA5;PPP2R1B;PTTG1;XPO6;RPS6KA2;STMN1;GRB10;AKT1;MAPK3;NDEL1;IER3;NUP214;NCOA1;H3F3B;DUSP1;MKL1;PRKCD;IL16;GAB2;CDC25C;ETV6;TNFRSF1A;PTPRE;SP1;CDK2;RAF1;METAP2
	LYN	1.5E-05	7.22E-04	-1.48	16.47	PPP1R15A;FASLG;ASAP1;CLEC9A;CBL;CSF2RA;MUC1;CASP7;PPP1R8;GRB10;CTLA4;TRIM69;PDE4A;MPZL1;MAPK3;MME;PRKCD;GAB2;CDC2;DOK3;FCGR2A;CDK2;BTK;RAF1;FCGR2C
	PCNA	3.4E-05	0.001	-1.70	17.47	CDKN1C;RFC5;FEN1;RFC4;PCNA;MME;FANCL;DSCC1;CDC25C;CDC2;CCNA2;CCNB2;PTP4A3;CCNB1;KIAA0101;EXO1;CHEK1;CDK2;TIMELESS;FSCN1
	TNIK	9.4E-05	0.004	4.65	-43.13	RANBP1;RRM1;GOT1;GLRX3;PDCD5;MRPL14;SOD2;TYMS;ACAT2;SLC9A1;UCHL1;MTHFD1;PSMA1;PSAT1;FSCN1;TXNL2;FKBP3
	PPP2R1A	0.00011	0.004	-1.64	14.99	CTNNBIP1;ARRB2;F11R;MLH1;PRKCZ;STRN4;HSPD1;PSMB6;KIAA1524;SGOL2;PSMA3;SGOL1;PSMA4;PSMB2;PSMA1;PPP2R1B;PRDX1;AKT1;MAPK3

* Chen, E.Y. et al. Enrichr: interactive and collaborative HTML5 gene list enrichment analysis tool. BMC Bioinformatics 14, 128 (2013). Kuleshov, M.V. et al. Enrichr: a comprehensive gene set enrichment analysis web server 2016 update. Nucleic Acids Res 44, W90-7 (2016). **Only the top 10 cell cycle-related proteins are shown; many more significant cell cycle-related proteins not shown.
