# Supplementary material for: Transcriptional blood signatures for active and amphotericin B treated visceral leishmaniasis in India
Source: PLoS Negl Trop Dis. 2019 Aug 16;13(8):e0007673. doi: 10.1371/journal.pntd.0007673 (PMC6713396; doi:10.1371/journal.pntd.0007673)

S1 Figure. Work flow for whole blood transcriptional expression profiling study of VL cases and controls used in the study. Numbers of DEGs represent the number of differentially expressed probes. The main text provides the number of genes encoded by these probes.

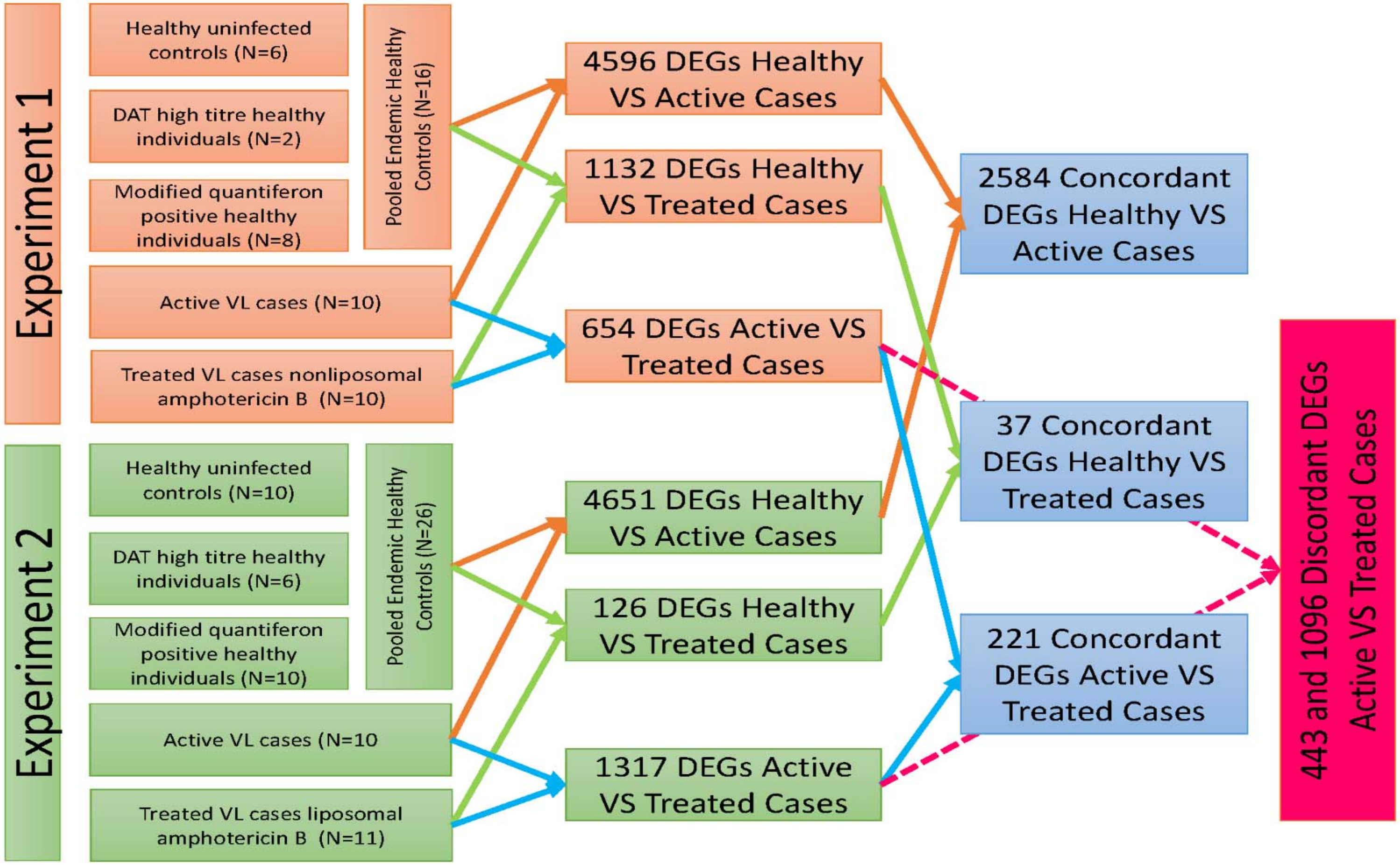

Supplement: S1 Fig — Numbers of DEGs represent the number of differentially expressed probes. The main text provides the number of genes encoded by these probes. (PDF) [file pntd.0007673.s010.pdf]
