# Supplementary material for: Transcriptional blood signatures for active and amphotericin B treated visceral leishmaniasis in India
Source: PLoS Negl Trop Dis. 2019 Aug 16;13(8):e0007673. doi: 10.1371/journal.pntd.0007673 (PMC6713396; doi:10.1371/journal.pntd.0007673)

S2 Figure. Schematic representation of Aryl Hydrocarbon Receptor (AHR) Signalling in Experiment 1. The diagram includes all AHR-interacting pathways generated in IPA using data for concordant differentially expressed ($P_{adj}<0.05$) genes across the two experiments. Molecules outlined in purple achieved fold-change >2. Genes in green have decreased expression in active cases compared to healthy controls, genes in red have increased expression. The more intense the colour the larger the fold change values. Expression values are based in experiment 1, representative of similar results obtained for concordant genes across the two experiments (see S3 Fig).

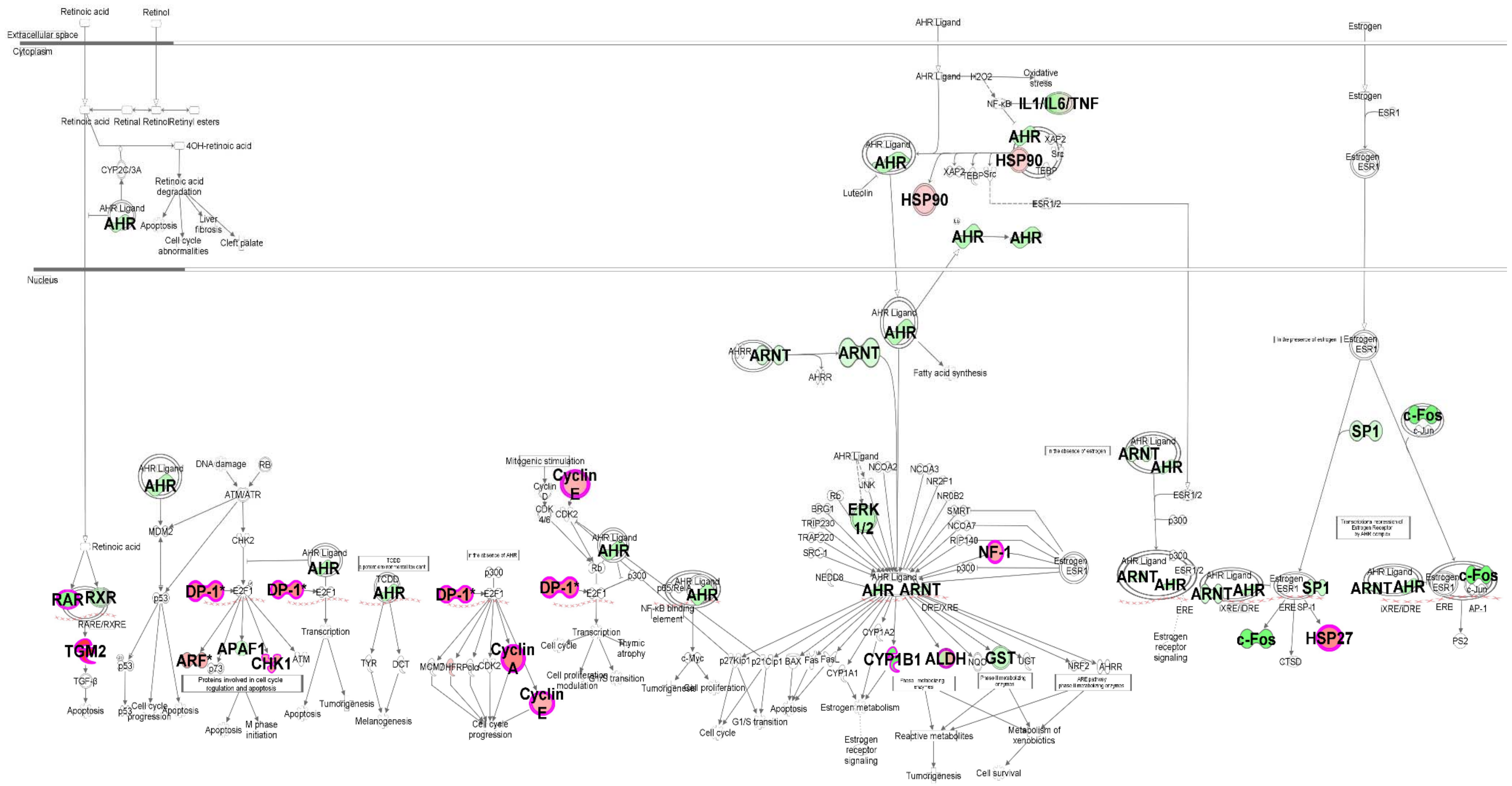

Supplement: S2 Fig — The diagram includes all AHR-interacting pathways generated in IPA using data for concordant differentially expressed (Padj<0.05) genes across the two experiments. Molecules outlined in purple achieved fold-change >2. Genes in green have decreased expression in active cases compared to healthy controls, genes in red have increased expression. The more intense the colour the larger the fold change values. Expression values are based in experiment 1, representative of similar results obtained for concordant genes across the two experiments (see S3 Fig). (PDF) [file pntd.0007673.s011.pdf]
